# Untargeted LC–HRMS of Dried Blood Spots Reveals Metabolic Alterations and Candidate Biomarkers in Glutaric Aciduria Type-1

**DOI:** 10.3390/metabo16030214

**Published:** 2026-03-23

**Authors:** Ahmed H. Mujamammi, Tagreed A. Mazi, Reem H. AlMalki, Essa M. Sabi, Maha Al Mogren, Meshari Alwazae, Randh AlAhmari, Khalid M. Sumaily, Rajaa Sebaa, Anas M. Abdel Rahman

**Affiliations:** 1 Clinical Biochemistry Unit, Pathology Department, College of Medicine, King Saud University, Riyadh 11461, Saudi Arabia; amujamammi@ksu.edu.sa (A.H.M.); ksumaily@ksu.edu.sa (K.M.S.); 2 Department of Community Health Sciences, College of Applied Medical Sciences, King Saud University, Riyadh 11451, Saudi Arabia; tmazi@ksu.edu.sa; 3 Metabolomics Section, Precision Medicine Laboratory Department, Genomics Medicine Center of Excellence, King Faisal Specialist Hospital and Research Centre (KFSHRC), Riyadh 11211, Saudi Arabia; 4 Computational Sciences Department, Genomics Medicine Center of Excellence, King Faisal Specialist Hospital and Research Centre (KFSHRC), Riyadh 11211, Saudi Arabia; alwazae@kfshrc.edu.sa; 5 Department of Medical Laboratories, College of Applied Medical Sciences, Shaqra University, Shaqra 11961, Saudi Arabia; 6 Department of Biochemistry and Molecular Medicine, College of Medicine, Alfaisal University, Riyadh 11533, Saudi Arabia

**Keywords:** glutarylcarnitine (C5DC), glutaric aciduria type-1 (GA-1), newborn screening (NBS), untargeted metabolomics, liquid chromatography–high-resolution mass spectrometry (LC–HRMS), dried blood spot (DBS)

## Abstract

**Background**: Glutaric aciduria type-1 (GA-1) is a genetic disorder caused by glutaryl-coenzyme A dehydrogenase deficiency, leading to the accumulation of glutaryl-CoA and its derivatives. Clinical manifestations include neurological abnormalities; however, the underlying pathological mechanisms remain unclear. Early diagnosis and intervention are crucial for minimizing adverse outcomes. To date, diagnostic methods have certain limitations, and there is a critical need for a sensitive biomarker for diagnosis. We aimed to characterize metabolic dysregulation and identify candidate biomarkers associated with GA-1 in biochemically confirmed patients compared to age- and sex-matched control subjects. **Methodology**: Untargeted metabolomics profiling of GA-1 patients (*n* = 29) was compared to matched control subjects by age and sex. Multivariate and univariate statistical analyses were performed to identify dysregulated metabolites. **Results**: Our findings revealed 220 endogenous human metabolites. Notably, there was a strong enrichment in carboxylic acids and derivatives, including amino acids and derivatives, hydroxy and keto acids, fatty acyls, sphingolipids, phosphatidylcholines, and nucleotides and nucleosides. Pathway analysis indicates alterations in the biosynthesis of cardiolipin and phosphatidylcholine, as well as in pyrimidine metabolism, the urea cycle, and amino sugar metabolism. We demonstrated a robust performance model for 6-Methylnonanoyl-CoA, displaying strong discriminative power. **Conclusions**: We identified broad dysregulation across various biochemical classes, reflecting an imbalance in energy metabolism that involves carbohydrate and lipid pathways. The results also highlight dysregulation in sphingolipids, phospholipids, and nucleotide metabolism. These findings are preliminary and the clinical relevance of these findings in patients with GA-1 requires further investigation. We identified candidate biomarkers capable of distinguishing GA-1 patients from controls; however, these findings require validation in independent cohorts.

## 1. Introduction

Glutaric aciduria type-1 (GA-1) is a rare inherited neurometabolic disorder characterized by defective catabolism of L-lysine, L-hydroxylysine, and L-tryptophan, estimated to affect approximately 1 in 110,000 people worldwide [1,2]. It results from a genetic deficiency of the enzyme glutaryl-CoA dehydrogenase (GCDH) [3], which leads to the buildup of glutaryl-CoA and its derivatives, such as glutaric acid (GA), 3-hydroxyglutaric acid (3-OHGA), and glutarylcarnitine (C5DC). This accumulation leads to a depletion of carnitine in body tissues and fluids [4,5]. Most undiagnosed and untreated GA-1 patients present between 3 and 36 months of age with an acute encephalopathic crisis, often triggered by physiological stress [4,5,6]. Clinical manifestations of the disease include macrocephaly and motor abnormalities (e.g., complex motor disorders, dystonia, dyskinesia, and hypotonia) with associated developmental impairment [7]. Clinical management of GA-1 involves a protein- and lysine-restricted diet and oral supplements such as L-carnitine and riboflavin [4,8].

The accumulation of neurotoxic metabolites, such as GA and 3-OHGA, is thought to drive acute and progressive neurological injury [9,10]. Repeated episodes of metabolic decompensation can lead to permanent neurological damage, as well as kidney failure and other systemic impairments [11]. Accordingly, early diagnosis and treatment are crucial to prevent disease progression, as they have been shown to lower the risk of adverse outcomes [8,12,13].

Current diagnostic methods used for GA-1 rely mainly on GA and 3-OHGA detection in urine [14], and measuring C5DC levels in blood [15]. However, both methods are limited by a high risk of false negatives, especially in mild- or low-excretor variants [2,16], which often exhibit normal or borderline-elevated biomarker levels, as we previously described [17]. Conversely, transient elevation of C5DC can also occur and may further complicate the diagnosis [17]. Although increased levels of C5DC and related ratios to other acylcarnitines (e.g., glutarylcarnitine/free carnitine (C5DC/C2), glutarylcarnitine/butyryl carnitine (C5DC/C4)) have been explored, none of these ratios were determined to be reliable markers [15]. Therefore, there is a critical need for a disease-specific and sensitive biomarker to improve diagnostic methods and support monitoring for GA-1 patients.

Untargeted metabolomics, primarily employing liquid chromatography–mass spectrometry (LC-MS) [18], offers promising capabilities for characterizing dysregulated metabolic pathways and identifying novel biomarkers associated with diseases. The metabolome, comprising small molecules such as amino acids, carbohydrates, and lipids, provides a dynamic biological snapshot that reflects phenotype-related changes [19]. Previous metabolomic profiling studies in GA-1 patients have identified several dysregulated metabolites, including pantothenic acid, betaine, 3-phosphoglycerate, inosine, and hypoxanthine, as well as sugars such as dextrose, ribose, and mannose [20]. A targeted metabolomic profiling, paired with machine learning, proposed a panel of metabolites, including classical disease markers, long-chain acylcarnitines, and their isobars, that discriminated GA-1 patients from those with other inborn errors of metabolism [21]. We have recently characterized metabolomic changes in newborns with false-positive GA-1 and transiently elevated C5DC levels and showed dysregulated altered sphingolipid and thiamine metabolism pathways with dysregulated levels of N-palmitoylcysteine, heptacarboxyporphyrin, 3-hydroxylinoleoylcarnitine, and monoacylglyceride (MG) (0:0/20:1/0:0) [17]. However, the broader metabolic background of GA-1 remains not fully understood, warranting further characterization.

Therefore, we conducted this study to compare the untargeted metabolomic profile of biochemically confirmed GA-1 patients under metabolic control with age- and sex-matched control subjects, aiming to characterize the metabolic dysregulation associated with GA-1 and identify potential biomarkers that can ultimately improve the accuracy and reliability of GA-1 screening and monitoring methods.

## 2. Materials and Methods

### 2.1. Study Participant and Ethical Approval

Dried blood spot (DBS) samples from biochemically confirmed GA-1 patients (*n* = 29) were categorized based on C5DC levels (Table S1) and compared to age- and sex-matched control subjects (*n* = 29). All samples were retrieved from the Metabolomics Section Laboratory—Precision Medicine Laboratory Department (PMLD) at the Genomics Medicine Center of Excellence (GMCoE), King Faisal Specialist Hospital and Research Center (KFSHRC), Riyadh, Saudi Arabia. This study was conducted in accordance with the Helsinki Declaration. The study protocol was reviewed and approved by the Institutional Review Board at KFSHRC (RAC #2160027). Samples were obtained as residual materials from routine clinical services, and patient consent was not required for their use.

### 2.2. Metabolomic Profiling

The GA-1-positive test was performed as part of DBS screening via an untargeted metabolomic approach, as reported elsewhere [17,22]. Analysis was conducted at the Metabolomics Section Laboratory—PMLD at GMCE, KFSHRC.

#### 2.2.1. Chemicals and Materials

LC-MS-grade acetonitrile (ACN), methanol (MeOH), formic acid, and water (H_2_O) were procured from Fisher Scientific (Ottawa, ON, Canada).

#### 2.2.2. Sample Preparation

Metabolites were extracted from DBS samples using a standard protocol previously reported [23]. In brief, a 3.2 mm punch was taken from each DBS, and metabolites were extracted by adding 300 µL of a solvent mixture (40:40:20 MeOH:ACN:H_2_O) and mixing with a ThermoMixer (Eppendorf, Hamburg, Germany) at 600 rpm for 2 h. at room temperature. Afterward, the samples were centrifuged at 16,000 rpm at 4 °C for 10 min, and the supernatants were transferred. Concurrently, additional punches from each sample were pooled to create QC samples, which were prepared to monitor system stability. Following extraction, the study and QC sample extracts were dried using a SpeedVac (Thermo Fisher, Braunschweig, Germany). The dried samples were then reconstituted in 100 µL of a mobile phase mixture (1:1) of phase A and phase B (A: 0.1% formic acid in water; B: 0.1% formic acid in (1:1 *v*/*v*) methanol: acetonitrile).

#### 2.2.3. LC–HRMS Metabolomics

Metabolic fingerprints were investigated using untargeted metabolomics, with samples analyzed by means of liquid chromatography–high-resolution mass spectrometry (LC–HRMS). The analysis utilized a Waters Acquity ultra-performance liquid chromatography (UPLC) system, coupled with a Xevo G2-S quadrupole time-of-flight mass spectrometer featuring an electrospray ionization source (ESI) (Waters Technologies, Milford, MA, USA), as previously described [24].

### 2.3. Data Processing

The MS raw data were processed using a standardized pipeline that involved peak alignment based on *m*/*z* values and retention times, peak picking, and signal filtering based on peak quality [25]. This process was carried out using the Progenesis QI software (version 3.0; Waters Technologies, Milford, MA, USA).

#### 2.3.1. Statistical Analysis and Metabolite Identification

Statistical analyses were performed using MetaboAnalyst (v.6.0; McGill University, Montreal, QC, Canada) (http://www.metaboanalyst.ca, accessed on 1 May 2024) [26] and The Mass Profiler Professional (MPP) software v.15.0 (Agilent Inc., Santa Clara, CA, USA). Before analysis, the data were median-normalized, Pareto-scaled, and log-transformed to achieve a normal distribution.

To reduce data dimensionality and examine whether GA-1 patients and the control group are distinguishable based on their metabolomic profiles, multivariate analyses were performed on normalized datasets. Partial least squares discriminant analysis (PLS-DA) was used as a supervised classification method to distinguish between patients with GA-1 and controls. Next, we performed orthogonal partial least squares discriminant analysis (OPLS-DA) to isolate predictive variation (associated with group discrimination) from orthogonal variation (unrelated noise or within-group variability). The model performance was evaluated using R^2^ (goodness of fit) and Q^2^ (predictive ability) metrics. To evaluate the risk of overfitting, model validity was assessed using permutation testing (100 permutations) [27].

To identify significant changes in the metabolomic profile between GA-1 patients and controls and to visualize the direction of these changes, univariate analysis was performed on normalized data. A volcano plot was generated using MPP software (v.15.0; Agilent Inc., Santa Clara, CA, USA). Fold change (FC) was calculated as the mean of (GA-1)/control, with a threshold FC cut-off of 1.5, an unpaired *t*-test was used to compare means, and differences were considered likely at Benjamini–Hochberg false discovery rate (FDR) correction *p* < 0.05. [28].

Metabolite annotation and identification were restricted to those compounds that were found significantly different between GA-1 patients compared to controls (FDR-adjusted *p* < 0.05, FC > 1.5) by volcano plot analysis. Metabolites were identified by acquiring accurate precursor masses, with theoretical MS/MS fragmentation tolerance set to 5 ppm for the Human Metabolome Database (HMDB) and 5 ppm for METLIN MS/MS (https://metlin.scripps.edu/, accessed on 1 May 2024). Metabolite identification levels follow the Metabolomics Standards Initiative (MSI) framework. Metabolite assignments in this study correspond to putative annotations (MSI Level 2) based on accurate mass and theoretical MS/MS fragmentation matching against public databases, without confirmation using authentic chemical standards. Fragmentations were filtered using in silico or empirical methods. Exogenous compounds, such as drugs, food additives, and environmental compounds, were manually excluded from the final list.

#### 2.3.2. Enrichment Analysis

To determine whether dysregulation(s) in specific metabolite classes are associated with GA-1 patients, metabolite set enrichment analysis (MSEA) was performed by chemical similarity (main class) based on the Global test [29] using MetaboAnalyst v6. (McGill University, Montreal, QC, Canada; http://metaboanalyst.ca, accessed on 1 May 2024) [30]. To include a broader set of metabolites and enable exploratory assessment of metabolite-class dysregulation, this analysis was based on annotated metabolites that were differentially dysregulated between GA-1 patients and controls in volcano plot analysis (FDR-adjusted *p* < 0.1, FC > 1.5). Metabolite classes were considered statistically significant when both *p* < 0.05 and FDR-adjusted *p* < 0.1.

#### 2.3.3. Pathway Analysis

Pathway analysis was performed employing MetaboAnalyst (McGill University, Montreal, QC, Canada; http://metaboanalyst.ca), accessed on 1 May 2024 [30]. To include a wider set of metabolites and enable exploratory assessment of broader pathway-level dysregulations, annotated metabolites that were differentially dysregulated between GA-1 patients and controls, identified through volcano plot analysis (FDR-adjusted *p* < 0.1, FC > 1.5), were compared against pathway-associated metabolite sets from The Small Molecule Pathway Database (SMPD). Fisher’s Exact test was used to assess over-representation, and the relative betweenness centrality was used for topology analysis. Pathways were considered statistically significant when both *p* < 0.05 and FDR-adjusted *p* < 0.1, whereas pathways only meeting the *p* < 0.05 criterion were classified as borderline.

#### 2.3.4. Potential Endogenous Biomarkers Evaluation

To evaluate the discriminatory power of metabolites differentially regulated by GA-1 in our samples, we employed PLS-DA analysis to discriminate between GA-1 patients and controls, as ranked by VIP scores, and examined selected individual metabolites. Receiver operating characteristic (ROC) curve analysis was performed using MetaboAnalyst (version 6.0; McGill University, Montreal, QC, Canada) (http://www.metaboanalyst.ca, accessed 1 May 2024) [26]. Monte Carlo cross-validation (MCCV) with balanced subsampling was employed to assess model stability. In each iteration, two-thirds of the data were used for training and estimating feature importance. Area Under the Curve (AUC) values with 95% confidence intervals (CIs) were computed for each model to assess performance, as well as classification metrics including sensitivity and specificity.

## 3. Results

### 3.1. Subject Characteristics

This analysis included samples from GA-1-positive patients (*n* = 29) and age- and sex-matched healthy controls (*n* = 29), with equal gender distribution across both groups. The mean age of the GA-1 group was (10.43 ± 9.5) years. Elevated blood C5DC levels biochemically confirm the diagnosis of GA-1. GA-1 group C5DC levels were (3.40 ± 2.3 µmol/L) compared to controls (0.09 ± 0.08 µmol/L) (*p* < 0.05). All GA-1 patients were undergoing active metabolic management. The demographics and clinical characteristics of study subjects are summarized in Table 1.

### 3.2. Distinct Metabolomic Alterations Characterized GA-1 vs. Control

A total of 16,872 ion features, comprising 10,925 and 5947 in the positive and negative samples, respectively, were detected in the study samples. After applying the filter by frequency with a cut-off percentage of 80 of all samples to remove missing values, 8635 features were retained and included in the statistical analysis.

**Figure 1:**
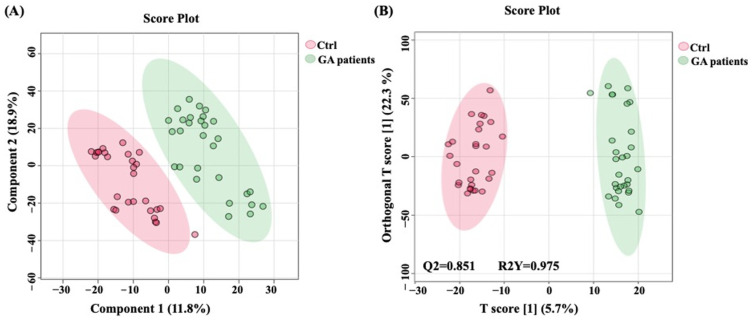
Supervised multivariate clustering models comparing GA-1 patients vs. the control group. (**A**) Partial least squares discriminant analysis (PLS-DA) based on 8635 ion features exhibits distinct clustering patterns, with GA-1 patients represented by “green” and age- and sex-matched controls by “red” ellipse. (**B**) The OPLS-DA model based on 8635 ion features showed clear separation between GA-1 patients, represented by the “green” ellipse, and age- and sex-matched controls, represented by the “red” ellipse. The clear separation between GA-1 patients and controls indicates distinctive metabolic profiles. Fitness of the model (R^2^ = 0.975) and predictive ability (Q^2^ = 0.851) values in a larger dataset (*n* = 100). Ctrl, control; GA-1, glutaric aciduria type-1. GA-1 patients (*n* = 29); control (*n* = 29).

To examine whether differences in metabolomic profile characterize GA-1 patients compared to controls, a PLS-DA was performed. Results show that GA-1 patients and controls exhibit distinct clustering patterns, highlighting unique metabolic changes associated with GA-1 that differentiate these patients from age- and sex-matched controls (Figure 1A). To further examine this divergence in metabolomic profile, accounting for potential covariance, OPLS-DA was performed. Results further confirm a distinct separation between the study groups (Figure 1B). The robustness of the models was evaluated by the fitness of the model (R^2^ = 0.975) and predictive ability (Q^2^ = 0.851), which suggests good predictive power. Results from permutation testing indicate that the model is statistically robust and not driven by random chance. The original model shows substantially higher R^2^ and Q^2^ values compared to the permuted models, confirming that the observed classification and predictive performance are significant and reliable, (Figure 1), supporting that the observed separation was unlikely to arise by chance.

**Figure 2:**
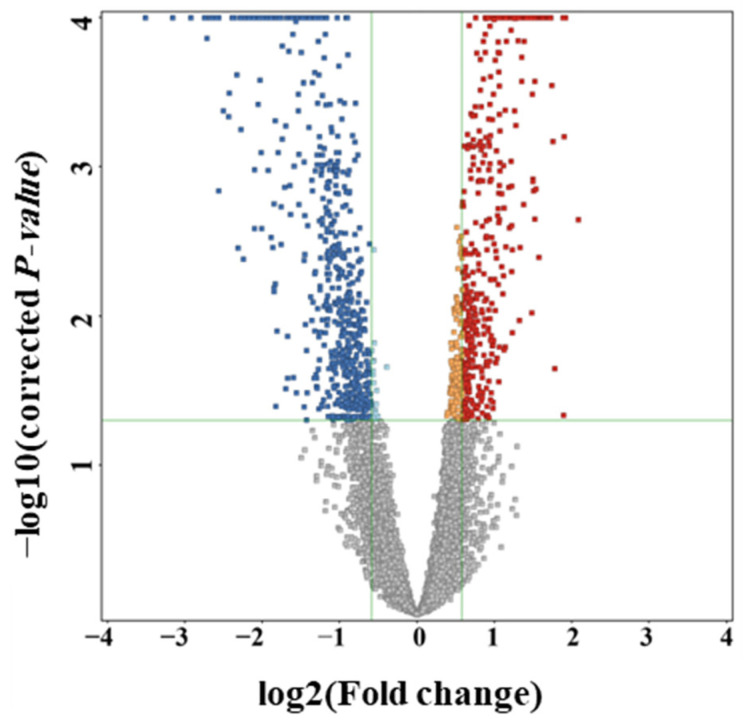
Volcano plot of differentially expressed metabolites between GA-1 patients and control group. The plot displays the results of unpaired two-tailed *t*-tests performed on 8635 ion features. Each metabolite is represented by a square. Color indicates the directionality of change: increase in GA-1 “red”; decrease in GA-1 “blue”; no change “grey”. Fold changes (FCs) calculated as (GA-1/control. The further the features from (0, 0), the more significant they are. Important features were selected using (unpaired *t*-test, FDR-adjusted *p* < 0.05, FC > 1.5). Both FC and *p*-value are log-transformed for analysis. Details are shown in Table S2.

Together, these findings highlight a distinct metabolic profile observed between GA-1 patients and controls. We further conducted a volcano plot analysis to identify significant changes in the metabolomic profile between GA-1 patients and controls and to visualize the direction of these changes (Figure 2). The results showed that 986 metabolites were significantly altered (FDR-adjusted *p* < 0.05, FC > 1.5), of which 413 were upregulated, and 573 were downregulated in GA-1 patients compared to healthy controls (Table S2).

Next, we annotated 555 significant ion features using accurate precursor masses and theoretical MS/MS fragmentation, matching them against the Human Metabolome Database (HMDB) and METLIN databases with a mass tolerance of 5 ppm. These assignments represent putative metabolite annotations derived from LC–HRMS data and are consistent with the untargeted metabolomics workflow described in Section 2.

A total of 220 endogenous metabolites were putatively annotated, including lipids and lipid-like molecules; nucleotides and nucleosides; amino acids, peptides, and analogs; carbohydrates and carbohydrate conjugates; coenzymes and cofactor derivatives; organic acids and derivatives; and hormones (Table S3 and S4).

### 3.3. Several Chemical Class Dysregulation Characterizes GA-1

To examine dysregulation in the metabolomic profile in GA-1 patients compared to controls, and to identify broader biochemical disruptions that may not be apparent through individual metabolites, chemical similarity enrichment analysis was performed (Table S5). Results indicate multiple metabolic classes are significantly enriched in GA-1 (Table S6). Among the most enriched were carboxylic acids and derivatives, which include amino acids (e.g., L-tyrosine, ornithine, D-glutamine, citrulline); amino acid derivatives; and peptides or amino acid conjugates. Additionally, this class includes glutathione derivatives.

Many hydroxy and keto acids and derivatives were also significantly enriched, along with organooxygen compounds. This class includes mainly carbohydrates and carbohydrate conjugates such as monosaccharides, sugar phosphates, and some derivatives.

GA-1 was also characterized by alterations in several sphingolipids, with dysregulation in several gangliosides, and in many fatty acyls, particularly dicarboxylic and hydroxylated fatty acids. Upon examining enrichment analysis by chemical subclass (Table S7), sphingolipids maintained strong enrichment. In addition, significant alterations (FDR-adjusted *p* < 0.1) were also observed across key subclasses of glycerophospholipids, particularly phosphatidylethanolamines (PEs) and their monoacyl forms, lysophosphatidylethanolamines (LysoPEs).

Nucleotides and nucleosides were also notably altered. This includes purine nucleotides, pyrimidine nucleotides, pyrimidine nucleosides, and ribonucleoside 3-phosphates. Some tetrapyrroles and derivatives, and steroid conjugates, were also dysregulated.

### 3.4. GA-1 Is Characterized by Altered Phospholipid, Nucleotide, and Urea Metabolism

**Figure 3:**
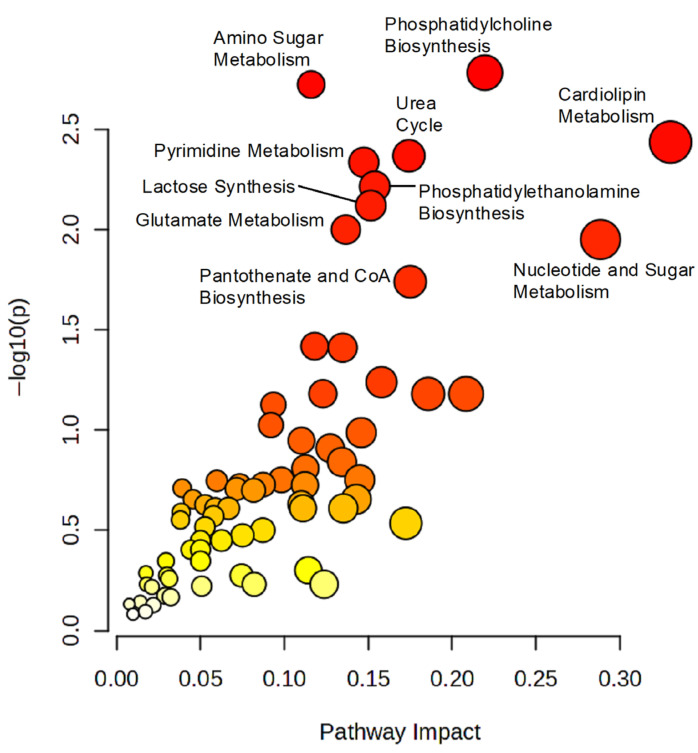
Pathway analysis of metabolites that significantly dysregulated metabolites in GA-1 patients vs. control group. Metabolic pathways differentially altered within GA-1 vs. control. Metabolites with differential alterations in GA-1 patients compared to controls (FDR-adjusted *p* < 0.1, FC > 1.5) were compared against pathway-associated metabolite sets from The Small Molecule Pathway Database (SMPD) [31]. Fisher’s Exact test was used to assess over-representation and the relative betweenness centrality was used for topology analysis. Significantly altered metabolic pathways are shown as nodes. The (*y*-axis) represents the *p*-values as determined by Fisher’s Exact test. The (*x*-axis) represents the impact of pathways as determined by the relative betweenness centrality–topology analysis. The size of the node represents the total number of hits. Detailed pathway analysis statistics are shown in Table S8.

To elucidate perturbations in metabolic pathways associated with GA-1, a pathway enrichment analysis was conducted, identifying several significantly altered pathways when comparing GA-1 patients to healthy controls (Figure 3) (Table S8).

Among the significantly dysregulated pathways (FDR-adjusted *p* < 0.1) with high topological impact are cardiolipin biosynthesis (impact = 0.298) and phosphatidylcholine biosynthesis (impact = 0.170), highlighting disrupted phospholipid metabolism. Additional pathways include pyrimidine metabolism (impact = 0.1985), urea cycle (impact = 0.1543), and amino sugar metabolism (impact = 0.1394). The combination of strong statistical impact scores suggests that these altered metabolites play central roles in GA-1.

This is followed by potentially relevant dysregulated pathways that showed borderline FDR-adjusted *p*-values and/or lower impact scores. These pathways include phosphatidylethanolamine biosynthesis (FDR-adjusted *p* = 0.1007; impact = 0), glutamate metabolism (FDR-adjusted *p* = 0.1234; impact = 0.1313), nucleotide sugars metabolism (FDR-adjusted *p* = 0.1234; impact = 0.5172), and ammonia recycling (FDR-adjusted *p* = 0.3206; impact = 0.1728). While not statistically significant after FDR adjustment, some of these pathways, i.e., nucleotide sugar metabolism and ammonia recycling, exhibited a high impact, indicating potential biological relevance in GA-1 that warrants further investigation. Finally, pathways such as pantothenate and CoA biosynthesis, lactose synthesis, and purine metabolism did not survive FDR correction (FDR > 0.18, impact < 0.1). Together, findings from pathway analysis highlight core dysregulated pathways in GA-1 mainly related to phospholipid, nucleotide, and amino sugar metabolism, with additional pathways of potential relevance that may contribute to the underlying metabolic disruption.

### 3.5. Exploring Potential Biomarkers for GA-1

**Figure 4:**
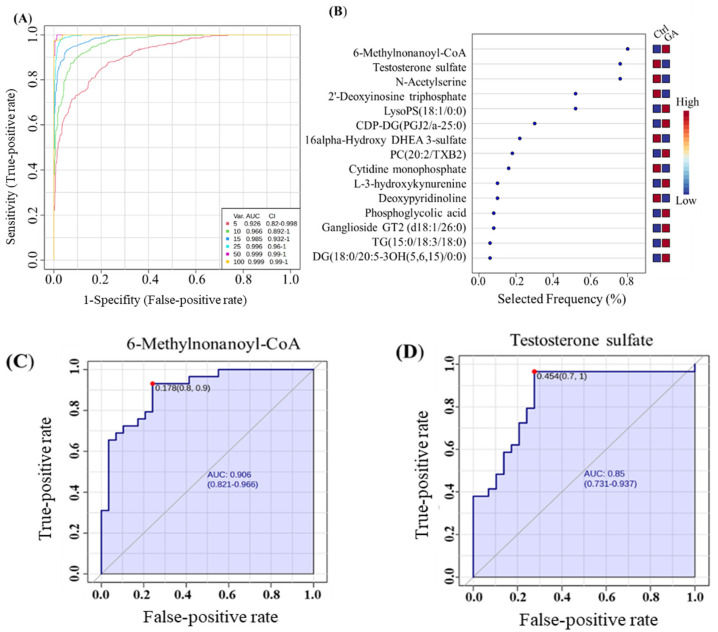

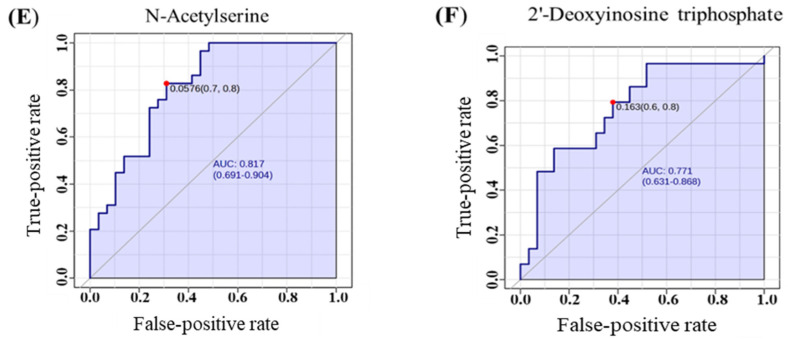
Classification performance of dysregulated endogenous metabolites in GA-1 patients vs. the control group. Based on 220 humans, endogenous metabolites significantly dysregulated metabolites in GA-1 patients vs. controls. (**A**) Receiver operating characteristics (ROCs) performance by the number of variables. (**B**) Variable importance plot and contribution to discrimination. A variable plot depicting the top 15 metabolites discriminating GA-1 patents vs. control. ROC performance curve for the top metabolites shown for (**C**) 6-Methylnonanoyl-CoA (AUC = 0.906) that was found upregulated in GA-1 vs. control; (**D**) testosterone sulfate (AUC = 0.85) that was found downregulated in GA-1 vs. control; (**E**) N-Acetylserine (AUC = 0.817), and (**F**) 2′-Deoxyinosine triphosphate (AUC = 0.771) that were found upregulated in GA-1 vs. control. Ctrl, control; GA-1, glutaric aciduria type-1.

To evaluate the diagnostic potential of dysregulated metabolites in distinguishing GA-1 patients from healthy controls, we employed discriminative metabolites between GA-1 patients and healthy controls based on VIP scores from PLS-DA analysis. The diagnostic performance of various models was evaluated using ROC curves, AUC values, and corresponding CI, as illustrated in Figure 4 and Table S9.

The models were tested using increasing numbers of top-ranked variables (5, 10, 15, 25, 50, and 100) and demonstrated progressive improvements in accuracy in differentiating between GA-1 patients and healthy controls (Figure 4A). The first model, utilizing five variables, achieved an AUC of 0.926 (95% CI: 0.82–0.998), indicating moderate discriminative power. The model with 10 variables performed similarly, with an AUC of 0.966 (95% CI: 0.892–1.000), indicating increased accuracy. As the number of variables increased, the models’ performance improved significantly, with the 25-variable model reaching an AUC of 0.996 (95% CI: 0.96–1). Both 50-variable and 100-variable models demonstrated near-perfect accuracy, with AUCs of 0.999 (95% CI: 0.99–1). Such findings suggest that even small subsets of key metabolites carry strong predictive value, with added variables refining classification confidence.

These results are further corroborated by the variable importance plot in (Figure 4B), which ranks the contribution of individual metabolites to the classification model, highlighting metabolites that are critical for group separation. These metabolites are likely to reflect core metabolic disruptions in GA-1 and establish the basis of high-performing models.

The top-ranked features consistently improve model performance as the number of variables increases, particularly when distinguishing between GA-1 and healthy controls. The high AUC values across all models, particularly those with 25 or more variables, indicate that the models exhibit excellent sensitivity and specificity, rendering them highly reliable for diagnostic purposes. Overall, the results indicate that increasing the number of variables in the models enhances their ability to accurately distinguish between the clinical groups. Models with 25 or more variables exhibit optimal performance, achieving nearly perfect classification between patients with GA-1 and healthy individuals.

Individual metabolites also demonstrated strong discriminative power. 6-Methylnonanoyl-CoA demonstrated a robust performance model, with an AUC of 0.906 (95% CI: 0.821–0.966), indicating strong discriminative power (Figure 4C). A notable threshold was observed at a false-positive rate (FPR) of 0.178 and a true-positive rate (TPR) of 0.9, indicating an effective balance between sensitivity and specificity. In contrast, testosterone sulfate showed moderate accuracy with an AUC of 0.85 (95% CI: 0.731–0.937) (Figure 4D). However, the red marker at an FPR of 0.454 and a TPR of 1.0 indicates a trade-off that favors sensitivity at the cost of a relatively higher FPR, which may limit its clinical utility. Similarly, N-Acetylserine exhibited the best overall performance with an AUC of 0.817 (95% CI: 0.691–0.904), and 2′-Deoxyinosine triphosphate (Figure 4E,F) performed exceptionally well, with an AUC of 0.771 (95% CI: 0.631–0.868). This compound maintained perfect sensitivity with negligible false positives, further supporting its diagnostic potential.

## 4. Discussion

The current work examined untargeted metabolomic profiling in a group of metabolically controlled GA-1 patients compared to age- and sex-matched control subjects. Our main findings indicate that GA-1 is associated with a distinct metabolomic profile characterized by (1) a broad dysregulation of pathways related to energy metabolism; (2) dysregulation in lipid metabolism, affecting mainly sphingolipids and phospholipids; (3) alterations in nucleotides and nucleosides metabolism; and (4) candidate metabolomic features that demonstrated strong classification of GA-1 from control within this exploratory dataset, supporting their prioritization for future validation studies.

Our group has recently examined metabolomic profiles in newborns with metabolic disorders and false-positive diagnoses of GA-1 showed transient elevations in C5DC, along with dysregulation in sphingolipid, phospholipid, and thiamine metabolism pathways [17]. In the current study, we expanded on these findings by characterizing metabolic alterations in a cohort of biochemically confirmed GA-1 patients. Our chemical enrichment analysis showed significant enrichment of carboxylic acids and derivatives as amino acids (e.g., L-tyrosine, ornithine, D-glutamine, citrulline), their derivatives, peptides, and amino acid conjugates, which highlights metabolic perturbations that extend beyond the classic dysregulation of lysine, hydroxylysine, and tryptophan metabolism, the hallmark of GA-1. In line with our results, data from in vitro studies show that GA-1 is associated with alterations in energy metabolism via mitochondrial dysfunction and defects in bioenergetic pathways including glycolysis, tricarboxylic acid cycle, and mitochondrial electron transport chain [32,33,34,35]. While our metabolomic data are consistent with these observations, the present study does not provide direct functional evidence of mitochondrial dysfunction.

The broader impact on energy homeostasis is also suggested by several enriched hydroxy and keto acids (e.g., malic acid, pyruvate, oxaloacetate, and α-ketoglutarate), which are central to carbohydrate metabolism through glycolysis, gluconeogenesis, and the tricarboxylic acid cycle, as well as the enrichment of multiple carbohydrate-related organooxygen compounds. Our data also suggest alterations in lipid metabolism, as evident by several enriched hydroxy and keto acids (e.g., 3-oxodecanoic acid, 3-oxododecanoic acid, 3-hydroxysebacic acid, 3-hydroxydodecanedioic acid), which are intermediates or byproducts of fatty acid β-oxidation, along with the dysregulations in the fatty acyl class, which include dicarboxylic acids and hydroxylated fatty acids; they suggest impaired fatty acid β-oxidation.

Findings from pathway analyses further corroborate a systemic dysregulation in energy metabolism, with dysregulated amino sugar metabolism, which highlights carbohydrate–amino acid metabolism crosstalk or, possibly, compensatory metabolic shift due to upstream dysregulation(s), glutamate metabolism, which is central to amino acid transamination, ammonia detoxification, and neurotransmitter cycling [36]. Previous experimental studies have suggested that chronic glutamate dysregulation may contribute to neurotoxicity [37,38]. In vitro evidence shows that elevated GA levels inhibit glutamate dehydrogenase activity, dysregulate glutamine degradation, and disrupt the normal neuron–astrocyte glutamate–glutamine cycle [39]. In this context, our findings may reflect metabolic alterations relevant to this neurotoxicity; however, given the observational design of this study, these findings need to be further explored in relation to neurodegenerative mechanisms.

We also observed an altered urea cycle and ammonia recycling, indicating impaired ammonia detoxification, and pantothenate and CoA biosynthesis, which is vital for amino acid metabolism, tricarboxylic acid cycle, and fatty acid oxidation [40]. The borderline significance for these pathways after FDR correction, in combination with the chemical enrichment analysis and pathway analysis, suggests potential relevance to GA-1. Together, our data demonstrate that GA-1, although traditionally classified as a disorder of protein catabolism, involves widespread dysregulation in protein, carbohydrate, and lipid metabolic pathways.

Also, our findings highlighted altered sphingolipid and phospholipid metabolism in GA-1. We observed alterations in sphingolipids, specifically gangliosides, which are glycosphingolipids highly abundant in the nervous system, vital in modulating bio-membrane function, and its dysregulation is associated with aging and neurodegenerative conditions [41].

Pathway analysis also showed dysregulated phospholipid metabolism with alterations in phosphatidylcholine, cardiolipin, and phosphatidylethanolamine biosynthesis. An altered phospholipid metabolism has implications for bio-membrane integrity function and plays a role in neurological disorders [42]. To our knowledge, sphingolipid and phospholipid profiling have not previously been reported in GA-1, and this study presents the first evidence of altered sphingolipid and phospholipid profiles associated with the disorder. The implications of such dysregulation to neurological manifestations require further investigation.

Multiple classes of nucleotides and nucleosides also showed significant enrichment. Among the dysregulations are: ADP which is central to cellular energy exchange [43], and its dysregulation is consistent with mitochondrial dysfunction and oxidative stress and impaired ATP regeneration [43]; nicotinamide ribotide, which is a precursor for nicotinamide adenine dinucleotide (NAD^+^) biosynthesis, which is central to energy metabolism and with roles in DNA repair, oxidative stress response and mitochondrial function [44]; and cytidine triphosphate, which is essential for RNA synthesis, phospholipid biosynthesis, and glycosylation [45]. We also observed enrichment in GMP and dADP, which are essential for nucleic acid synthesis, signaling, and energy metabolism. Results from pathway analysis support findings from enrichment analysis and show significantly dysregulated pyrimidine metabolism, with borderline significance observed for nucleotide sugar metabolism. These findings reflect broad dysregulation in purine- and pyrimidine-derived nucleotide and nucleoside metabolism, which may reflect impaired mitochondrial dysfunction and energy homeostasis, disruption in nucleic acid metabolism, and altered cellular signaling.

To date, a sensitive, disease-specific biomarker for GA-1 is yet to be identified. Our analysis identified several potential candidate biomarkers with promising classification performance in this exploratory analysis. We show that 6-Methylnonanoyl-CoA demonstrated a robust performance model, with an AUC of 0.906 (95% CI: 0.821–0.966), indicating strong discriminative power. However, these findings require further validation in independent cohorts using targeted approaches.

Considering the established GA-1 biomarkers such as glutarylcarnitine, glutaric acid, 3-hydroxyglutaric acid and glutaconic acid identified in this untargeted metabolomics dataset, we bear in mind that their clinical relevance and diagnostic utility should be assessed independently from their levels recorded in the context of the routine targeted biochemical investigations summarized in Table 1. Such variations are largely due to differences in timing, sample collection conditions, and detection limits associated with the particular analytical instrument(s) used in these analyses. A key strength of this study is the inclusion of biochemically confirmed GA-1 cases across diverse age groups and biological sexes. However, the limited sample highlights the need for further validation. Importantly, samples were obtained from metabolically controlled patients undergoing active clinical management. Therefore, the observed metabolomic profile likely reflects the treated phenotype rather than the untreated disease. This raises the possibility that metabolomic profiling may have future utility not only for biomarker discovery but also for monitoring metabolic control and treatment response in GA-1 patients. Due to the lack of detailed clinical characterization of neurological symptoms in the study cohort, the implications of the observed metabolic dysregulations in the pathophysiology of GA-1 and neurological manifestations remain to be elucidated and warrant further investigation. Future studies incorporating targeted metabolomics will be essential for establishing biological mechanisms and validating biomarkers.

## 5. Conclusions

We performed untargeted metabolomic profiling on samples from biochemically confirmed GA-1 patients. Our results, supported by both enrichment and pathway analyses, show that GA-1, while primarily a disorder of amino acid metabolism, also involves broader disruptions in energy metabolism, including carbohydrate and lipid pathways. It also reveals dysregulation in the metabolism of sphingolipids, phospholipids, and nucleotides. However, these findings should be considered for hypothesis generating and require validation in independent cohorts using targeted approaches. The clinical significance of these findings in relation to the GA-1 phenotype, disease progression, and treatment response requires further investigation. We propose several candidate metabolomic features, including a compound putatively annotated as 6-Methylnonanoyl-CoA, as potential biomarkers for distinguishing GA-1 patients from healthy controls. These findings should be interpreted as exploratory metabolomic observations rather than validated biomarkers and require confirmation in larger cohorts using targeted metabolomics.

## Figures and Tables

**Table 1:** Demographic characteristics of study subjects.

	GA-1	Control	*p*-Value
*n* (F/M)	29 (15/14)	29 (15/14)	
Age (Years)	10.43 ± 9.5	10.4 ± 11.3	0.9875
C5DC (µmol/L)	3.40 ± 2.3	0.09 ± 0.08	3.81 × 10^−10^

Data are presented as mean ± SD. Comparisons were performed by *t*-test. CD5C, glutarylcarnitine; GA-1, glutaric aciduria type-1.

## Data Availability

The original contributions presented in this study are included in the article/Supplementary Material. Further inquiries can be directed to the corresponding author.

## Supplementary Materials

The following supporting information can be downloaded at: https://www.mdpi.com/article/10.3390/metabo16030214/s1. Table S1: Demographic data, as well as information on medication and supplement use, were recorded for the study participants. Table S2: Volcano Plot Analysis of GA-1 Patients vs. Controls. Fold change (FC) was calculated as the mean of GA-1/Control, with a threshold FC cut-off of 1.5. Unpaired *t*-test was used to compare group means, and statistical significance was determined false discovery rate (FDR)-adjusted *p*-values < 0.05 and a fold change (FC) threshold of 1.5. A total of 986 features were significantly altered, including 413 upregulated and 573 downregulated in GA-1 patients. Table S3: Annotation of metabolites identified 555 compounds significantly altered in GA-1 patients versus controls (FC ≥ 1.5; FDR-adjusted *p* < 0.05, unpaired *t*-test), including 220 endogenous and 335 exogenous metabolites, based on open-source databases. Table S4: Annotation of metabolites identified significantly altered in GA-1 patients versus controls using in-house Library. Table S5: List of Endogenous Metabolites Included in Chemical Similarity Enrichment Analysis of GA-1 Patients vs. Controls. Metabolites were selected based on statistical significance in volcano plot analysis (FDR-adjusted *p* < 0.5, FC > 1.5). Metabolite Set Enrichment Analysis (MSEA) was then performed by chemical similarity (main class). Table S6: Metabolite Set Enrichment Analysis (MSEA) Performed by Chemical Class Enrichment Analysis comparing GA-1 Patients vs. Controls. Details of chemical similarity enrichment analysis, identifying metabolite classes significantly overrepresented among the dysregulated metabolites in GA-1. For each class, the total number of metabolites in the background set, expected count under the null hypothesis, actual number of significant hits. Significant enrichment was considered at FDR-adjusted *p* < 0.1. Table S7: Metabolite Set Enrichment Analysis (MSEA) Performed by Chemical Sub-Class Enrichment Analysis comparing GA-1 Patients vs. Controls. Details of chemical similarity enrichment analysis, identifying metabolite sub-classes significantly overrepresented among the dysregulated metabolites in GA-1. For each class, the total number of metabolites in the background set, expected count under the null hypothesis. Significant enrichment was considered at FDR-adjusted *p* < 0.1. Table S8: Pathway Enrichment Analysis comparing GA-1 Patients vs. Controls. Details of actual number of significant hits, raw *p*-values, Holm-adjusted *p*-values, and false discovery rate FDR-adjusted *p*-values are reported. Significant pathway enrichment was considered at FDR-adjusted *p* < 0.1. Table S9: Variable Importance Plot and Contribution to Discrimination. A variable plot depicting the top 15 metabolites discriminating GA-1 patients vs Control.

## Abbreviations

3-OHGA, 3-hydroxyglutaric acid; ACN, Acetonitrile; ADP, Adenosine diphosphate; CI, Confidence intervals; dADP, Deoxyadenosine diphosphate; dIDP, Deoxyinosine diphosphate; DBS, Dried blood spot; ESI, Electrospray ionization source; FPR, False-positive rate; FDR, False discovery rate; FC, Fold change; GA, Glutaric acid; GA-1, Glutaric aciduria type-1; GCDH, Glutaryl-CoA dehydrogenase; C5DC, Glutarylcarnitine; C5DC/C4, Glutarylcarnitine/butyryl carnitine; C5DC/C2, Glutarylcarnitine/free carnitine; GMP, Guanosine monophosphate; KFSHRC, King Faisal Specialist Hospital and Research Center; LVs, Latent variables; LC–HRMS, Liquid chromatography–high-resolution mass spectrometry; LC-MS, Liquid chromatography–mass spectrometry; LysoPCs, Lysophosphatidylcholines; MPP, Mass Profiler Professional; MS, Mass spectrometry; MSEA, Metabolite set enrichment analysis; PMLD, Precision Medicine Laboratory Department; MeOH, Methanol; MG, Monoacylglyceride; MCCV, Monte Carlo cross-validation; NAD^+^, Nicotinamide adenine dinucleotide; OPLS-DA, Orthogonal partial least squares discriminant analysis; PLS-DA, Partial least squares discriminant analysis; PCs, Phosphatidylcholines; PEs, Phosphatidylethanolamines; ESI+ and ESI−, Positive and negative electrospray ionization modes; QC, Quality control; QTOF, Quadrupole time-of-flight; ROC, Receiver operating characteristic; GMCoE, The Genomics Medicine Center of Excellence; HMDB, The Human Metabolome Database; SMPD, The Small Molecule Pathway Database; TPR, True positive rate; UPLC, Ultra-performance liquid chromatography; VIP, Variable Importance in Projection; H_2_O, Water.
